# Deciphering the Molecular Variations of Pine Wood Nematode *Bursaphelenchus xylophilus* with Different Virulence

**DOI:** 10.1371/journal.pone.0156040

**Published:** 2016-05-25

**Authors:** Xiaolei Ding, Jianren Ye, Sixi Lin, Xiaoqin Wu, Dewei Li, Bo Nian

**Affiliations:** 1 Co-Innovation Center for Sustainable Forestry in Southern China, College of Forestry, Nanjing Forestry University, Nanjing, Jiangsu, China; 2 Jiangsu Key Laboratory for Prevention and Management of Invasive Species, Nanjing, Jiangsu, China; 3 The Connecticut Agricultural Experiment Station Valley Laboratory, Windsor, Connecticut, 06095, United States of America; INRA, FRANCE

## Abstract

*Bursaphelenchus xylophilus* is the causative agent of pine wilt disease which has caused huge economic losses in many countries. It has been reported that two forms of pine wood nematodes existed in its native region, i.e., with strong virulence and weak virulence. However, little is known about the molecular differences between the two forms. To better understand their molecular variations, transcriptome and genome sequences of three strongly virulent and one weakly virulent strains were analyzed. We found 238 transcripts and 84 exons which showed notable changes between the two virulent forms. Functional analyses of both differentially expressed transcripts and exons indicated that different virulence strains showed dissimilar nematode growth, reproduction, and oxidoreductase activities. In addition, we also detected a small number of exon-skipping events in *B*. *xylophilus*. Meanwhile, 117 SNPs were identified as potential genetic markers in distinguishing the two forms. Four of them were further proved to have undergone allele specific expressions and possibly interrupted the target site of evolutionary conserved *B*. *xylophilus* miR-47. These particular SNPs were experimentally verified by including eight additional strains to ensure the validity of our sequencing results. These results could help researchers to better diagnose nematode species with different virulence and facilitate the control of pine wilt disease.

## Introduction

Pine wood nematode (PWN), *Bursaphelenchus xylophilus*, is the causative agent of pine wilt disease (PWD) [1] and native to North America where it usually only damages exotic pine trees. It has spread to Asian and European countries such as Japan, China, South Korea, and Portugal [2]. It has a complex life cycle which involves beetles, pine trees, fungi, bacteria and causes enormous economic losses every year [3]. Although there are several hypotheses about the mechanism of PWD, such as cellulase (it was possible that the destruction of pine cells might be triggered by cell wall degrading enzymes such as cellulase), phytotoxin (some experiments showed that phytotoxins isolated from *B*. *xylophilus* can directly cause wilt symptoms) and terpenoid hypotheses (some scientists believe that cavitation and xylem water column breakage of pine tree are caused by terpenoids), the pathogenic mechanism of PWD still remains to be elucidated [4–7]. It is reported that two forms of PWN existed in its native region, i.e., strongly virulent (SV) and weakly virulent (WV) [8, 9]. Usually, the virulence of PWN was evaluated by an inoculation test and Takemoto et al. (2005) reported another classification method based on PCR-RFLP patterns of heat-shock protein 70A [10]. Some previous studies proved that the lower reproductivity and increased developmental time of a generation were observed in weakly virulent strains rather than strongly virulent strains [11, 12]. Other studies indicated PWN with different virulence contained different enzymatic and non-enzymatic molecules which were involved in oxidative stress metabolism [13]. In the early stage of PWD, PWN has to fight with various plant immune responses. The initial host reaction to nematode invasion would be an oxidative burst [14, 15]. Also, high virulence isolates of *B*. *xylophilus* could withstand higher H_2_O_2_ concentrations in comparison with low virulence *B*. *xylophilus* [16]. Thus, the different oxidative abilities between the two forms could contribute to their virulence variation. Besides those reproductive and biochemical differences, limited information was found to describe the genetic variations between these two forms on a genomic scale. Thus, it is necessary to perform a genome-wide study on the two forms to gain insights on their genetic differences and to explore new ways for accurate virulence detection.

Since the first draft of the *B*. *xylophilus* genome was released in 2011, researchers have investigated this nematode on the genome level [3]. With the help of high-throughput sequencing, we were able to perform analyses on isoform alteration, SNP identification and allele-specific expression [17, 18]. A recent published paper focused on comparative transcriptome analysis between *B*. *xylophilus* and *B*. *mucronatus* indicated the transcriptome variations between these two close species. Meanwhile, genome wide SNP identifications proved the SNP diversity among different *B*. *xylophilus* populations [19, 20]. Another study also reported numerous novel parasitism genes which may be crucial for the mediation of interactions of *B*. *xylophilus* with its host using comparative transcriptomics [21]. Besides those studies focused on gene expression and SNP changes, it would also be interesting to observe if any allele-specific expression had existed in *B*. *xylophilus* since allele-specific expression can control gene expression and interruption of the regulation process could lead to disease [22, 23]. In this study, high-throughput RNA and DNA sequencing were used together to perform genome-wide analyses on *B*. *xylophilus* with different virulence. We selected and sequenced four nematode strains with different virulence to detect molecular differences between the two forms. Moreover, another eight nematode strains were included as additional experimental materials to better verify our sequencing results.

Generally, we found that different virulent strains exhibited different exons and transcript expression and that these changes mainly involved nematode growth, reproductivity and oxidoreductase activities. Also, we have selected and verified a subset of potential SNP markers for virulence detection.

## Materials and Methods

### PWN strains and virulence test

*B*. *xylophilus* strains AA3 and AMA3 from Anhui province, ZL1 from Zhejiang province, and YW4 from Yunnan province were used for next-generation DNA and RNA sequencing. Other additional strains used for experimental verification included: SG4 (Sichuan province), SC2 (Shandong province), AN5 (Anhui province), HE2 (Hubei province), GZ1 (Guizhou province), AW5 (Anhui province), JC1 (Jiangsu province), and JL1 (Jiangsu province). All these nematode strains were maintained in the Forest Protection Laboratory, Nanjing Forestry University and the virulence of these strains were demonstrated by previous inoculation tests on pine trees as described by Aikawa et al. [24]. Two-year-old *Pinus thunbergii* seedlings under similar growing conditions were used in inoculation studies. *P*. *thunbergii* stems were cut at 15 cm above the soil level with a sterilized scalpel. A piece of sterile absorbent cotton was placed over each wound and 200 μL nematodes suspension (about 2,000 individuals) was pipetted into wounds. Then the wounds were covered by parafilm. Control consisted of *P*. *thunbergii* seedlings inoculated with 200 μL sterile deionized water. The disease development of *P*. *thunbergii* seedlings was observed at an interval of three days. Each treatment had six replicates. The disease severity of *P*. *thunbergii* seedlings was divided into five levels: 0, the seedlings remaining healthy with green needles and growing well; I, a few needles turning brown; II, half of the needles turning brown and the terminal shoots of seedlings bending; III, most of the needles turning brown and dead and the terminal shoots of seedlings drooping; IV, all of the needles turning brown and the whole seedling wilted. The disease severity index was calculated below:

$$\text{Disease severity index}\;(\text{DSI})=\frac{\Sigma \;number\;of\;disease\;plants\times symptom\;stage}{Total\;number\;of\;plants\times highest\;symptom\;stage}\times 100$$

All nematode strains were cultured on the fungus *Botrytis cinerea* at 25°C for almost one week. Nematodes were extracted with a Baermann funnel [25, 26]. After 12 h, nematodes were collected at the bottom of the funnel and stored in water suspension using a 1.5 ml centrifuge tube.

### DNA and RNA extraction

Total RNA was extracted using Trizol reagent following the product descriptions (https://www.lifetechnologies.com/order/catalog/product/15596026). Genomic DNA was isolated from the *B*. *xylophilus* strains using the CTAB method [27]. Both DNA and RNA qualities were assessed by Nanodrop 2000 (http://www.nanodrop.com/Productnd2000overview.aspx) and visualized in agarose gel. Four DNA and RNA libraries for each strain were constructed and sequenced using Illumina platform un-stranded 100bp paired-end protocol. 8G DNA-seq and RNA-seq were obtained separately for each nematode strain (Novogene, Beijing).

### ITS rDNA amplification and RFLP analysis

From a practical aspect, *B*. *xylophilus*, when isolated from infected trees, always contained more than one nematode species, i.e, *B*. *mucronatus*. In order to prove that the nematodes used here were not contaminated with *B*. *mucronatus*, an ITS-PCR-RFLP method was used to distinguish the two forms with one *B*. *mucronatus* strain as the negative control. A segment of rDNA was amplified by PCR according to Iwahori H’s method [28]. PCR was carried out in a 50 μl reaction mixture containing 2 μl of nematode DNA, 25 μl pre-mix, 19 μl sterilized distilled water and 2 μl forward and reverse primers. The procedure for PCR was as follows: 94°C for 2 min, followed by 40 cycles (94°C for 1 min, 55°C for 30 sec, and 72°C for 1 min), and finally 72°C for 10 min. The PCR product from each isolate was digested with 3 U of restriction endonucleases AluI, HaeIII, HinfI, MspI and RsaI according to the instructions provided by the manufacturer (Takara, Dalian). Restriction products were analyzed by electrophoresis on a 2% agarose gel.

### Data analysis

All raw sequencing data were first assessed by Fastqc (http://www.bioinformatics.babraham.ac.uk/projects/fastqc/) to ensure the sequencing quality. Adapters were then removed by cutadapt (https://cutadapt.readthe-docs.org/en/stable/). Filtered reads were used for subsequent analyses.

#### Alignment, assembly and annotation using RNA-seq

Genome alignment and transcript assembly processes were done by following the protocol described by Cole Trapnell [29]. Firstly, RNA-seq data from YW4, AA3, AMA3 and ZL1 were aligned to the genome using Tophat allowing two mismatches (v2.0.10) [30]. Secondly, four transcripts sets were generated by Cufflinks using the frag-bias-correct option [31]. Thirdly, four transcripts were merged together into one final *B*. *xylophilus* transcriptome assembly using Cuffmerge based on genome data. This non-redundant transcriptome assembly was used as reference assembly for all the rest of our analyses. Previously assembled transcripts were annotated using Blast2Go by searching against nr, Gene Ontology and Pfam databases [32, 33]. GO enrichment analysis of differentially expressed transcripts and exons were performed by agriGO analysis tool kit based on Blast2Go annotation result (Pvalue<0.05) [34]. *De novo* assembly for each nematode strain was carried out using Trinity software (http://trinityrnaseq.github.io/).

#### Expression profiling of transcripts and exons

Differentially expressed transcripts were identified using cuffdiff and DESeq (Pvalue<0.05) [31, 35]. Self-developed R script was used to display general expressions of all transcripts. Hierarchy cluster analysis was made using MEV package (http://www.tm4.org/mev.html). Differentially expressed exons were identified and visualized by DEXSeq (Pvalue<0.05) and visualized using plotDEXSeq [36]. VennDiagram package (http://cran.r-project.org/web/packages/-VennDiagram/index.html) was used to show overlapped events. The expressions of DE transcripts were confirmed by quantitative RT-PCR using SYBR Premix Ex Taq (Takara, Dalian, China) according to the manufacturers’ instruction on 7500 real-time system (Applied Biosystems, Carlsbad, CA, USA) [37]. The results were normalized to the expression level of the *B*. *xylophilus Actin* gene (GenBank: EU100952). Relative expression levels were calculated using the $2_{\;T}^{-\Delta \Delta C}$method [38]. All RT-PCR experiments were done with three replicates.

#### Exon skipping identification

Putative exon skipping events were identified by using GESS from RNA-seq data and each skipping event was recorded in gff3 format compatible with MISO. Thus, expressions of exon skipping events were evaluated by MISO (exon-centric” analysis) using GESS output results [39, 40]. Exon skipping events were visualized using Sashimi plot (http://miso.readthedocs.org/en/fastmiso/sashimi.html). Upstream and downstream primers were designed based on our assembly results for downstream PCR validation [41]. PCR was carried out by the following procedure: 94°C for 2 min, followed by 37 cycles (94°C for 30 sec, 58°C for 30 sec, and 72°C for 30 sec), and finally 72°C for 10 min. The PCR products were examined by 1% agarose gel electrophoresis.

#### DNA-seq analysis process

Sequencing reads were aligned to the *B*. *xylophilus* genome using Burrows-wheeler Aligner [42]. Duplicates were removed by Picard (http://broadinstitute.github.io/picard/). Putative SNPs and Indels were called by samtools mpileup with–ugBDS option and freebayes, respectively [43]. Then, VCFtools was applied to remove Indels and generate genotypes matrix. Population splitting tree was generated by Treemix with default parameter [44]. Selected SNPs sites were validated by both PCR and clone sequencing. PCR was carried out by the following procedure: 94°C for 2 min, followed by 35 cycles (94°C for 30 sec, 50°C for 30 sec, and 72°C for 30 sec), and finally 72°C for 10 min. All PCR products were then cloned into a pMD19-T vector (Takara, Dalian) and sent for sequencing (20 clones per SNP sites).

## Results

The virulence of all *B*. *xylophilus* strains were evaluated by inoculation tests (see materials and methods). 34 days after inoculation, 6 strains (AW5, YW4, GZ1, JL1, HE2, JC1) showed DSI values lower than 50 while another 6 strains (SG4, SC2, AN5, AMA3, AA3, ZL1) showed DSI values higher than 50 (S1 Table). The enzyme digestion results indicated that all *B*. *xylophilus* strains shared the same band distributions which were different from the negative control (S1 Fig). Therefore, all *B*. *xylophilus* strains used in this study were not contaminated with other nematode species. Thus, three SV *B*. *xylophilus* strains (AMA3, DSI: 100; AA3, DSI: 87.5; and ZL1, DSI: 79.2) with highest DSI values and one WV strain (YW4, DSI: 4.2) with lowest DSI value were selected to perform transcriptome and genome sequencing while other additional strains were used for experimental validation.

### Genome based transcript assembly and annotation

After barcode removal and quality control, ~8G paired end data with 100bp read lengths per sample were obtained. Overall, 78% of the RNA-seq data were successfully aligned to the *B*. *xylophilus* genome. Then, a non-redundant transcript assembly was generated with 20,465 transcripts derived from 17,199 genes. The length distributions of assembled transcripts mostly ranged from 100bp to 2.5kb, and seven transcripts were identified with their length over 20kb (S2A Fig). A *de novo* assembly approach using RNA-seq alone was also applied in this research to evaluate our assembly result. The transcript lengths of *de novo* assembly were obviously shorter than the genome based method and YW4 assembly was poorly assembled without the reference genome (S2A Fig).

Transcripts annotations indicated that only 40% of the assembled transcripts (8304) could be assigned with known functions. The most enriched GO terms were nematode development, protein binding and hydrolase activity (S2B Fig).

### Identification of differentially expressed transcripts

**Fig 1:**
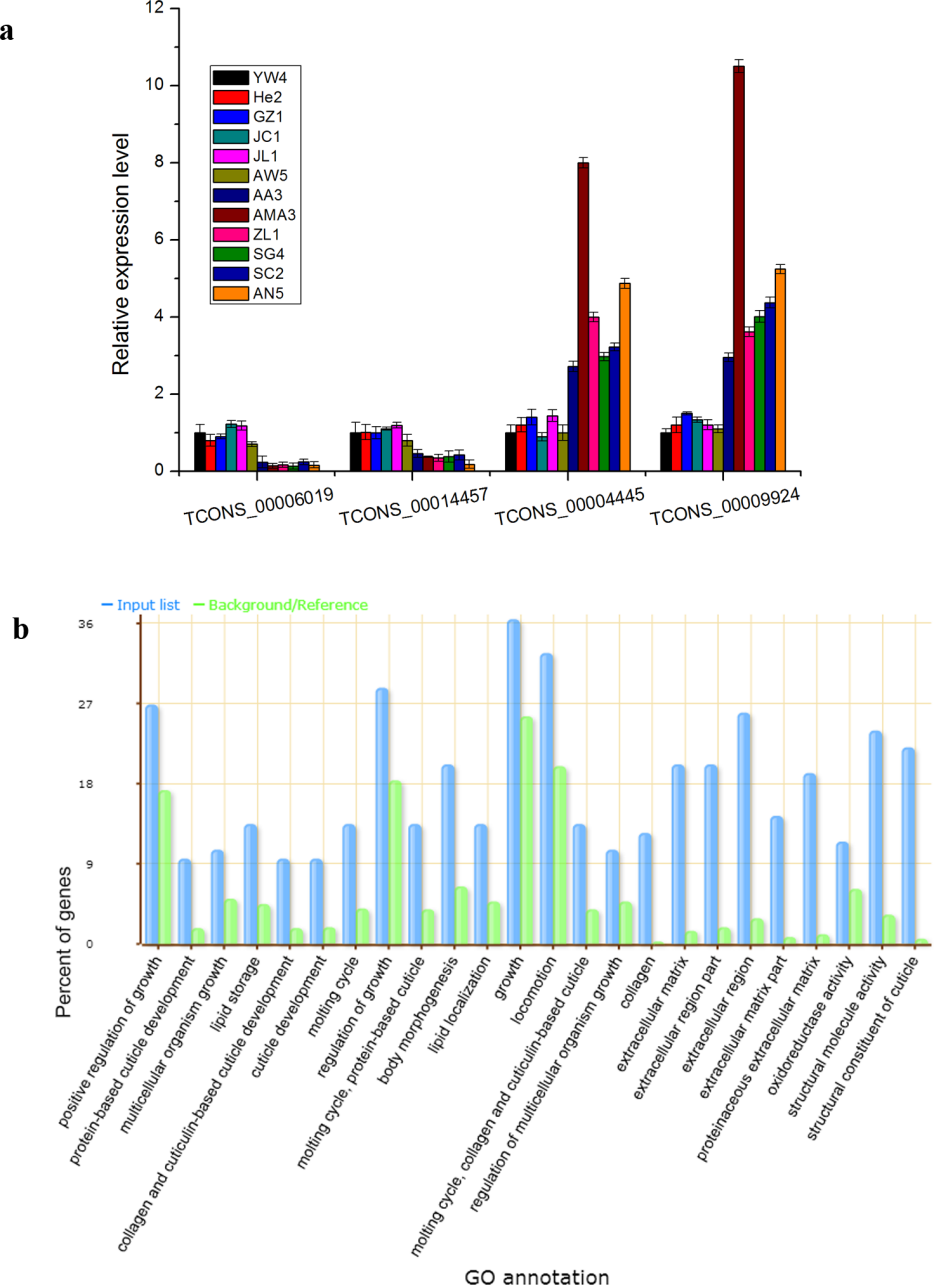
Validation and GO enrichment analysis of differentially expressed transctipts. (a) qPCR validation of four selected transcripts. (b) GO enrichment results of differentially expressed transcripts (Pvalue<0.05).

Two popular gene quantification packages, Cuffdiff and DESeq, were applied to detect differentially expressed (DE) transcripts [29, 45]. Finally, 238 overlapped DE transcripts were identified using the two packages together (Pvalue<0.05). To better supported our findings, additional strains (DSI>50: SG4, SC2, AN5; DSI<50: HE2, GZ1, AW5, JC1, JL1) were also included in qPCR validation of four up and down regulated transcripts (Fig 1A). The results suggested that up regulated transcripts TCONS_00004445 and TCONS_00009924 showed higher expression levels within SV strains. As for down regulated transcripts, TCONS_00006019 and TCONS_00014457 from WV showed more than two times higher expressions than those from SV strains. Other additional strains with DSI<50 shared the same expression patterns as WV strains while strains with DSI>50 showed the same expression patterns as SV strains. Functional annotation revealed that DE transcripts were mainly involved in nematode reproduction, development, and oxidoreductase activities (Fig 1B). The hierarchy clustering results of DE transcripts showed that SV strains were grouped together rather than the WV strain (S3 Fig).

### Differentially expressed exon and exon-skipping event detection

**Fig 2:**
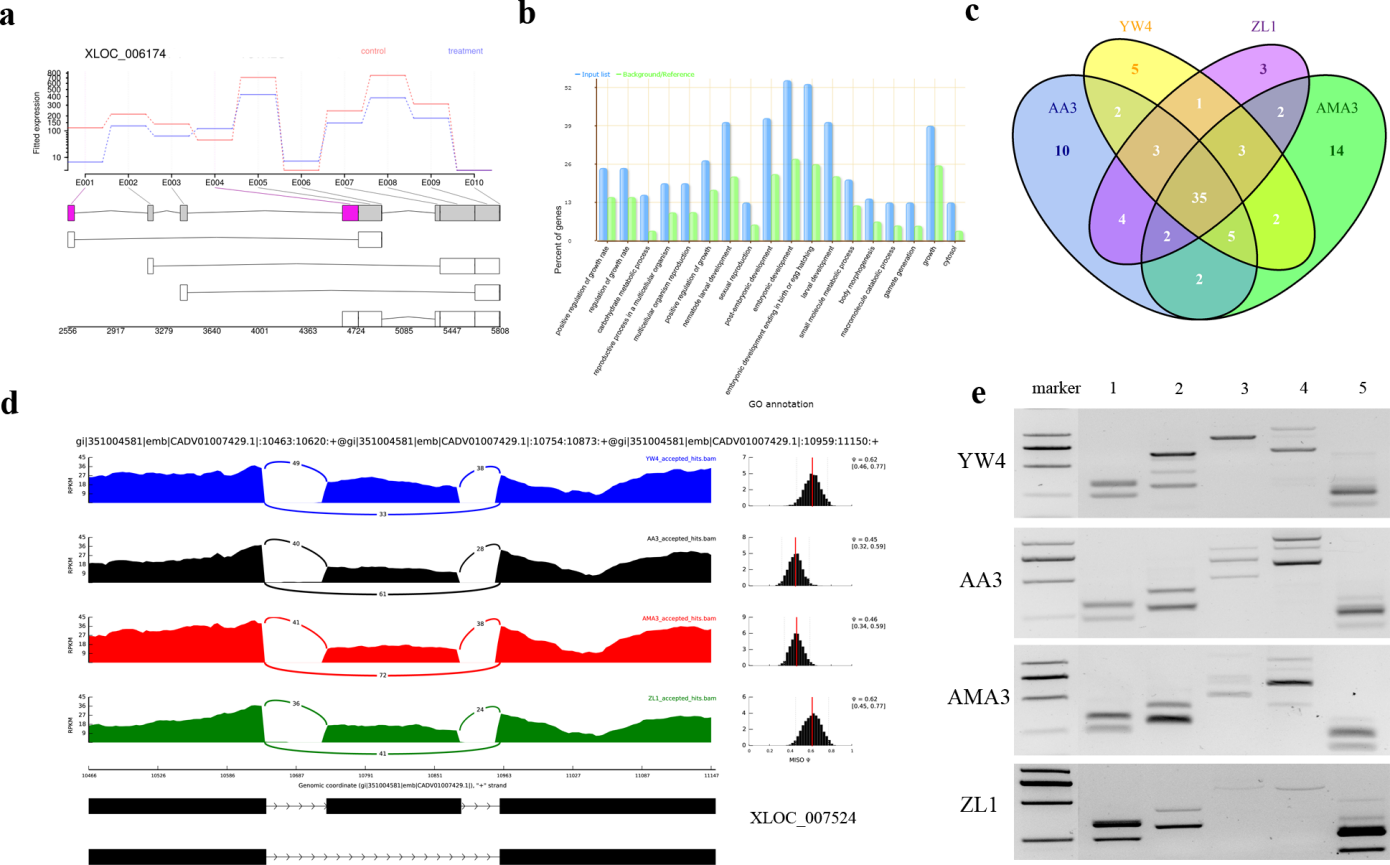
Exon-skipping events and differentially expressed exons found in *B*. *xylophilus*. (a) Visualization of differentially expressed exons in *Bx-eng-1* gene (highlighted in red). (b) GO enrichment results of differentially expressed exons (P-value<0.05). (c) Venn-diagram showing the predicted exon-skipping events. (d) Sashimi plot of exon-skipping event in XLOC_007524. (e) Experimental validation of exon-skipping events using PCR amplification. (lane 1 indicated exon-skipping events found in XLOC_002568, lane 2: XLOC_007524, lane 3: XLOC_002051, lane 4: XLOC_001151, lane 5: XLOC_000101)

Here, we tried to use RNA-seq data to observe the expression change of certain exon and exon-skipping event [36]. Expression profiling of exons revealed that there were 75 genes containing 84 DE exons (S2 Table). For instance, XLOC_006174, known as *Bx-eng-1* gene was not identified as a DE gene but contained two DE exons (Fig 2A). GO enrichment analysis indicated transcripts contained those DE exons were mainly associated with functions like: growth rate regulation and larval development (Fig 2B).

Meanwhile, 63, 65, 53 and 56 exon-skipping candidates were identified from AA3, AMA3, ZL1 and YW4, respectively, using RNA-seq alone (Fig 2C). Among them, 35 recurrent exon-skipping candidates were detected in all nematode strains. Detailed information for all identified exon-skippings was presented in S3 Table and visualized using sashimi plot (Fig 2D). Unfortunately, we were not able to find any DE exon-skipping events after comparing SV with WV strains. Five exon-skipping events from XLOC_002568, XLOC_007524, XLOC_002051, XLOC_001151 and XLOC_000101) were validated by PCR amplification (Fig 2E).

### Screening for SNPs and allele specific expressions

**Fig 3:**
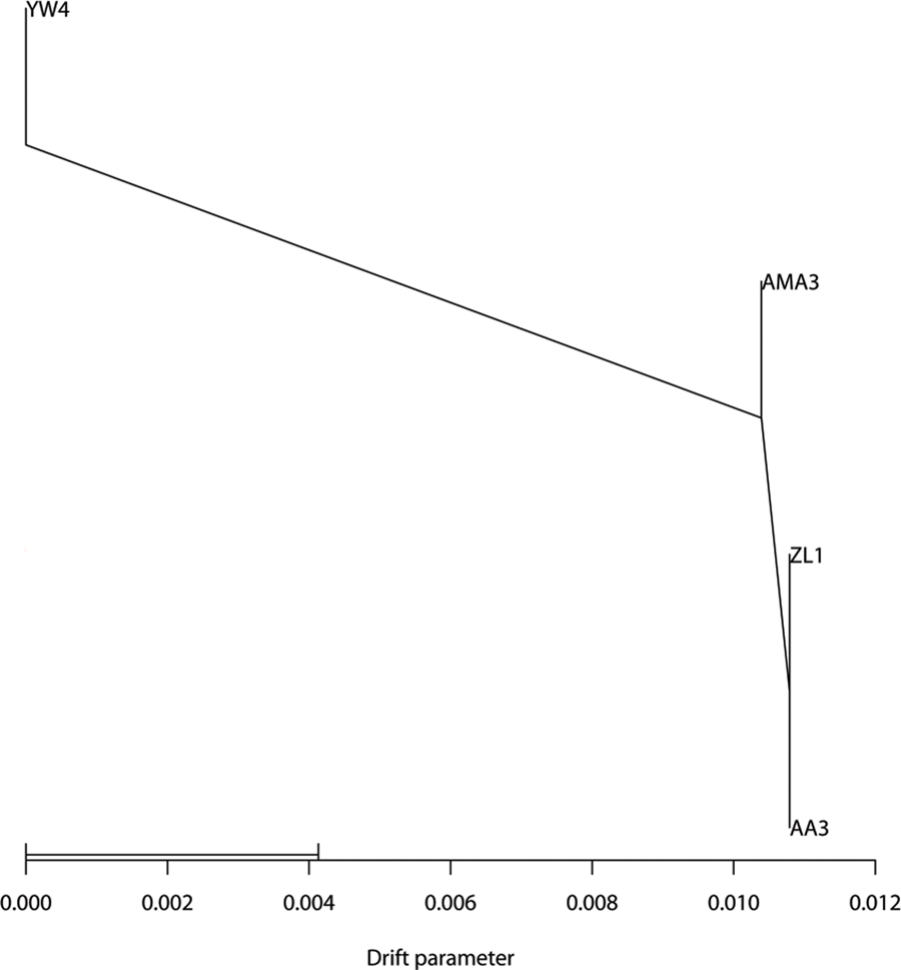
Population splitting tree generated by Treemix based on SNP allele frequency.

**Fig 4:**
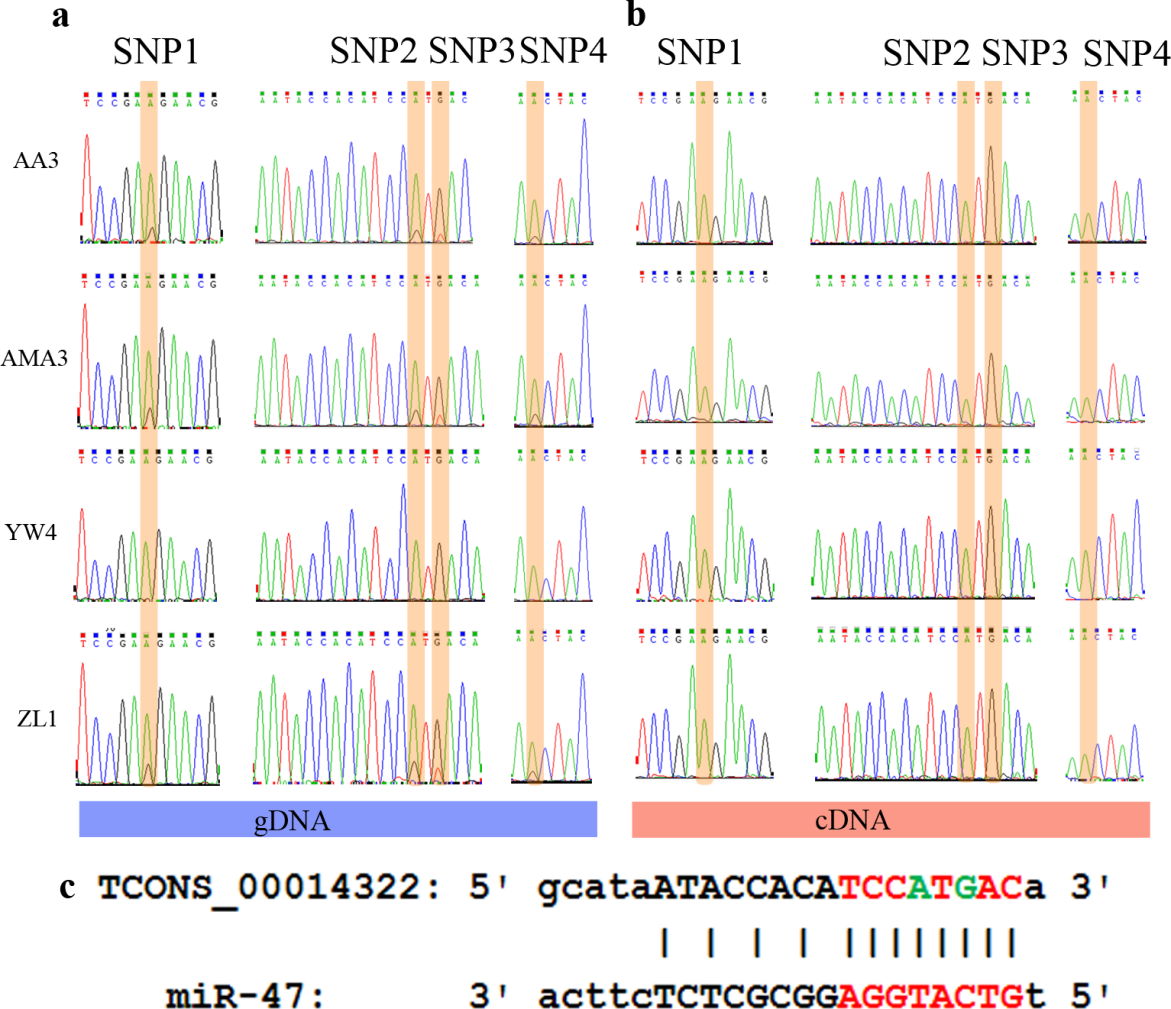
Sanger sequencing results of four SNPs with allele specific expressions found in *B*. *xylophilus*. (a) Sequencing chromatograms of SNPs sites amplified from genomic DNA (SNPs sites are highlighted in yellow) (b) Sequencing chromatograms of SNPs sites amplified from cDNA (allele specific expressions are highlighted in yellow). (c) miR-47 targeting region interrupted by allele specific expressions, (red indicated the seed region of miR-47, green indicated the nucleotides underwent allele specific expressions).

**Fig 5:**
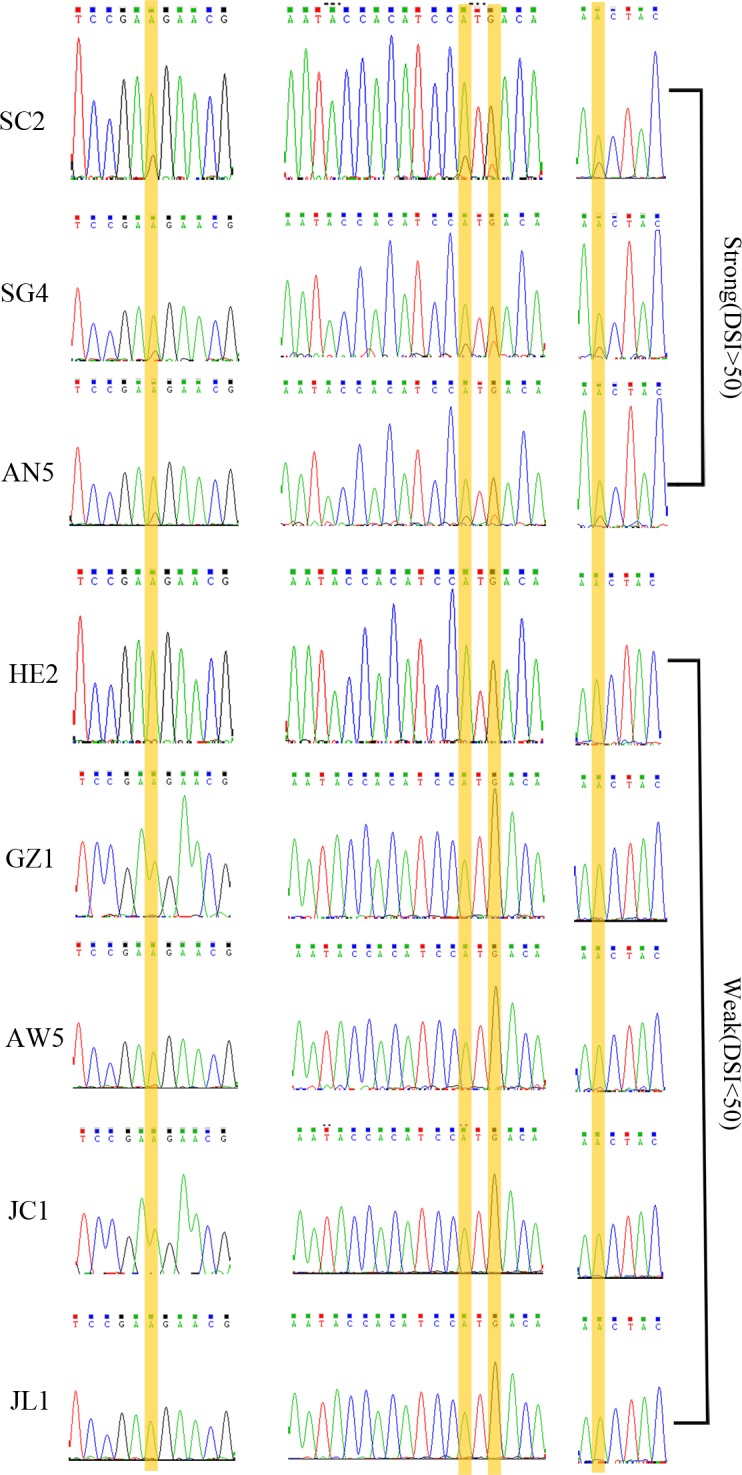
Sanger sequencing results of additional strains indicated the validity of SNPs identification. (SNPs are highlighted in yellow).

Samtools (http://samtools.sourceforge.net/) and freebayes (https://github.com/ekg/freebayes) packages were used together for searching SNPs to ensure the reliability of the results. Finally, 310,014 overlapped SNPs sites were detected after merging the output from these two packages. Among them, 82,577 (26.6%) of SNPs were located in exons while 227,437 (73.4%) were located in introns or other unannotated regions. The population splitting tree generated based on SNP allele frequency provided a good support for all samples used in this study. WV strains were separated from SV strains (Fig 3). After that, 117 SNPs were selected as possible markers to distinguish these two forms based on genotype variations. These 117 SNPs shared uniform genotypes among SV but exhibited other genotypes in WV. In addition, 45 of them were located in exons and 72 of them located in introns (S4 Table). Particularly, we found there were four SNPs which could undergo allele specific expressions since the genome variations were not found in corresponding transcripts (Fig 4A and 4B, Table 1). Moreover, those allele specific expressions would affect the target reconition of miR-47 which was identified in our previous research (Fig 4C) [46]. Theoretically, it would be more reasonable to test those markers with additional *B*. *xylophilus* strains. Thus, we included additional strains to ensure the prediction by amplifying and sequencing those particular four SNPs from both genome and transcriptome levels. Both PCR and clone sequencing were used to verify SNPs sites (Figs 4A and 5, S4 Fig), while PCR sequencing alone was used to prove the corresponding allele specific expressions using cDNA (Fig 4A and 4B). All sequencing results indicated that additional *B*. *xylophilus* strains with DSI <50 shared the same genotypes as WV strains while *B*. *xylophilus* strains with DSI >50 shared the same genotypes as SV strains.

**Table 1:** List of four experimentally verified SNPs markers.

SNP ID	Genomic loci	Genotypes				Alternate base
		AA3	AMA3	YW4	ZL1	
**SNP1**	gi|351006313|emb|CADV01005697.1|:13329	1	1	0	1	A->G
**SNP2**	gi|351006313|emb|CADV01005697.1|:13366	1	1	0	1	A->G
**SNP3**	gi|351006313|emb|CADV01005697.1|:13368	1	1	0	1	G->T
**SNP4**	gi|351006313|emb|CADV01005697.1|:13418	1	1	0	1	A->G

0 for invariant allele, 1 for Heterozygote.

## Discussion

All subsequent analyses on DE transcripts, DE exons and splicings required reliable transcript assembly. Previous research on the *B*. *xylophilus* transcriptome reported a transcript assembly comprised 23,765 mRNA contigs with maximum length of 5,847 using *de novo* methods [19]. Compared with the early *de novo* assembly, the genome-based transcript assembly reduced the transcripts number to 20,465 and increased the maximal length to 24,372 (S2A Fig). In addition, there were 904 transcripts with lengths longer than 5847. Successful amplifications of predicted transcripts and exon-skipping events demonstrated the accuracy of our assembly (Figs 1A and 2E). Consequently, this transcript assembly could be widely used for relevant research on *B*. *xylophilus*.

Previous study reported that the virulence level of *B*. *xylophilus* can be estimated by investigating the reproductive and growth ability on *B*. *cinerea* or in branch sections of *P*. *thunbergii* trees [11]. Consequently, it is reasonable to find that DE transcripts were mainly involved with growth regulation (Fig 1B, S5 Table). This discovery supported the fact that different nematode virulence strains may have different growth abilities. Meanwhile, oxidoreductase activity was identified as another enriched function in DE transcripts (Fig 1B). Recent studies indicated that more than ten anti-oxidant proteins were identified in the secretome of *B*. *xylophilus*. The secreted anti-oxidant enzymes would play an important role in protecting *B*. *xylophilus* itself from oxygen free radicals in the pine tree [3, 47]. Another study also proved that different virulent *B*. *xylophilus* exhibited different tolerance to oxidative stress which is crucial component in host defense mechanisms [16]. Thus, the up regulation of DE transcripts, for instance: TCONS_00004445 and TCONS_00009924, suggested a more dynamic oxidoreductase activity within SV strains and this was in line with the above conclusions (Fig 1A, S5 Table). In addition, up-regulated transcript TCONS_00010273 was annotated as Proteinase inhibitor I29 which had been widely detected in the *B*. *xylophilus* secretome [47]. It contained inhibitors of cysteine peptidases while the plant cysteine peptidases were involved in the regulation of the defense system against pathogenic microbes and nematodes [48, 49]. Therefore, the I29 inhibitor could possibly target the plant cysteine peptidases and fight against the plant defense system. Higher expression of this transcript may facilitate the pine tree infection process in SV strains.

Transcripts are composed of exons and expressions of exons could be more complicated [50]. The differential usage of exons between these two forms could cause the change of protein sequences and thus lead to certain functional changes. However, descriptions on DE exons seemed to have been missed in previous research on *B*. *xylophilus*. Here, we found that most DE exons were found within transcripts annotated as nematode growth regulation and reproductive ability (Fig 2B). The result was also consistent with the conclusion that different growth rates were found between *B*. *xylophilus* strains with different virulence. Furthermore, we also attempted to detect around 60 exon-skipping events which had not been reported before (Fig 2C). Unfortunately, we did not observe any DE exon-skipping evens between different virulence strains, but it could be a new perspective to investigate the functional changes of the PWN. We may get some interesting results with the help of deeper sequencing coverage or even more complete assembled and annotated genome. Nevertheless, it could be a new perspective to study this important parasite.

One of the crucial applications of DNA-seq is to investigate genetic variations between individuals using whole-genome sequencing [51]. Meanwhile, SNPs are favorable markers for many applications in population ecology, evolution and conservation genetics [52]. In this research, a total of 310,014 SNPs were found in *B*. *xylophilus* [3]. Similar research on pine wood nematode SNPs detection in Japan detected many more SNPs sites than our results [20]. This could be explained by different strains used for analysis since we used nematode strains isolated from China rather than Japan. Also, quite stringent parameters used in our SNP detection pipeline could be another main reason for the reduction of SNPs (see material and methods for detail). Despite that, both of the studies proved that more than 95% of the SNPs were homozygous and more than 60% of the SNPs were located within intergenic and intron regions. Importantly, we reported 117 potential SNPs markers to classify *B*. *xylophilus* strains with different virulence after comparing the genotypes of SV and WV strains. However, it seemed that the level of diversity in the *B*. *xylophilus* genome was high but it had been observed in other hyper-diverse organisms. Previous studies indicated that organisms with large population size, short generation times, small body sizes were more likely to be hyper-diverse[53]. *B*. *xylophilus* was known to have all aforementioned features and it was possible to find some sequence polymorphisms among different *B*. *xylophilus* populations in a local area. Future study should be focused on using more nematode strains to calibrate our results. However, those potential markers will provide another possible way for researchers to evaluate nematode virulence other than using traditional inoculation tests.

Here, four allele specific expressions located in TCONS_00014322 were observed for the first time in SV strains. Interestingly, the seed region of miR-47 was complementary to 3’ region of TCONS_00014322 while two allele specific expressions also resided in this target region (Fig 4C). It is known that the RNA-induced silencing complex recognized their target messages based on perfect complementarity between the miRNAs and their target genes [54]. Consequently, these two allele specific expressions ensured that miR-47 could successfully target TCONS_00014322 (Fig 4C). Possibly, those allele specific expressions may alter the recognition site of miRNA to conduct post transcriptional regulation.

## Conclusions

Our results demonstrated that the *B*. *xylophilus* SV and WV strains showed dissimilar expression patterns on both the transcripts and exon levels. The most significant variation between the two forms was observed in growth, reproduction and oxidoreductase activities. The establishment of putative SNPs markers will provide potential methods to distinguish these two nematode forms on the molecular level. This will assist researchers to better diagnose this nematode species and facilitate the control of pine wilt disease.

## Supporting Information

S1 Fig ITS-PCR-RFLP analysis of the *B*. *xylophilus* and *B*. *mucronatus* strains. (marker: DL 1000)
(TIF)

S2 Fig A general overview of *B*. *xylophilus* transcript assembly and annotation. (a) Length distributions of different assembly methods. (b) Blast2go annotation results of *B*. *xylophilus* transcript assembly.
(TIF)

S3 Fig Hierarchy clustering of *B*. *xylophilus* differentially expressed transcripts. (TIF)

S4 Fig Clone sequencing of *B*. *xylophilus* strains for SNPs verification. (TIF)

S1 Table Disease severity index of *B*. *xylophilus* based on *P*. *thunbergii* inoculation tests. (XLSX)

S2 Table Detailed information of differentially expressed exons generated by DEXSeq. (PDF)

S3 Table An overview of all exon-skipping events based on RNA-seq data. (XLS)

S4 Table Genomic coordinates of SNPs which showed different genotypes between strongly and weakly virulent strains. (XLS)

S5 Table GO enrichment analysis of differentially expressed transcripts and exons. (XLSX)
