# Supplementary figures and images for: Deciphering the Molecular Variations of Pine Wood Nematode Bursaphelenchus xylophilus with Different Virulence
Source: PLoS One. 2016 May 25;11(5):e0156040. doi: 10.1371/journal.pone.0156040 (PMC4880305; doi:10.1371/journal.pone.0156040)

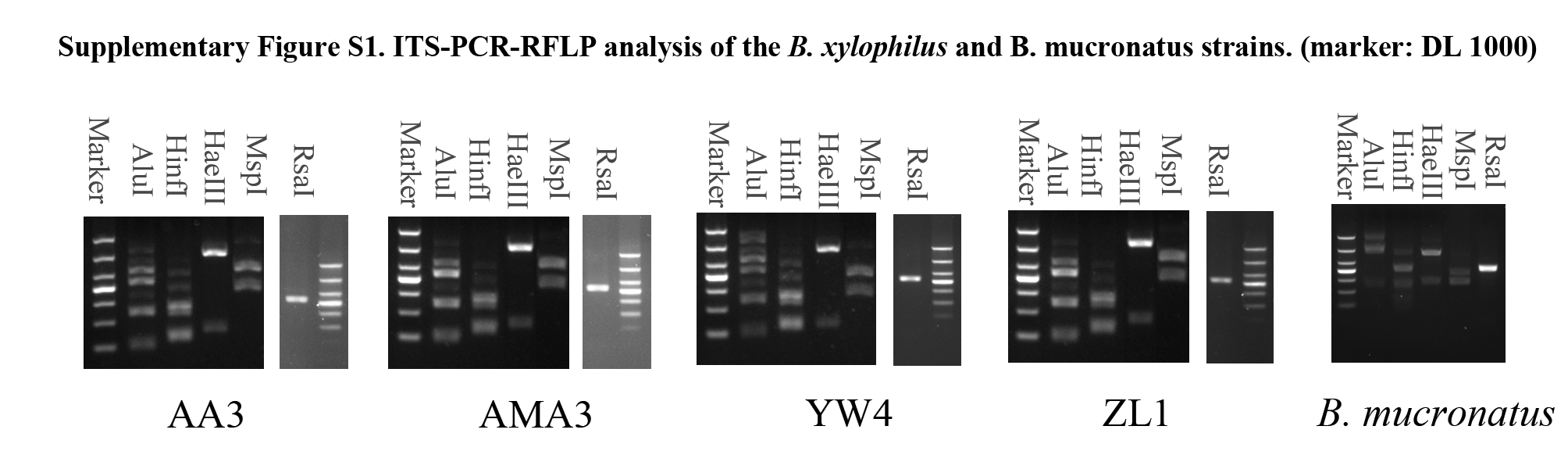

Supplement: S1 Fig — (marker: DL 1000) (TIF) [file pone.0156040.s001.tif]

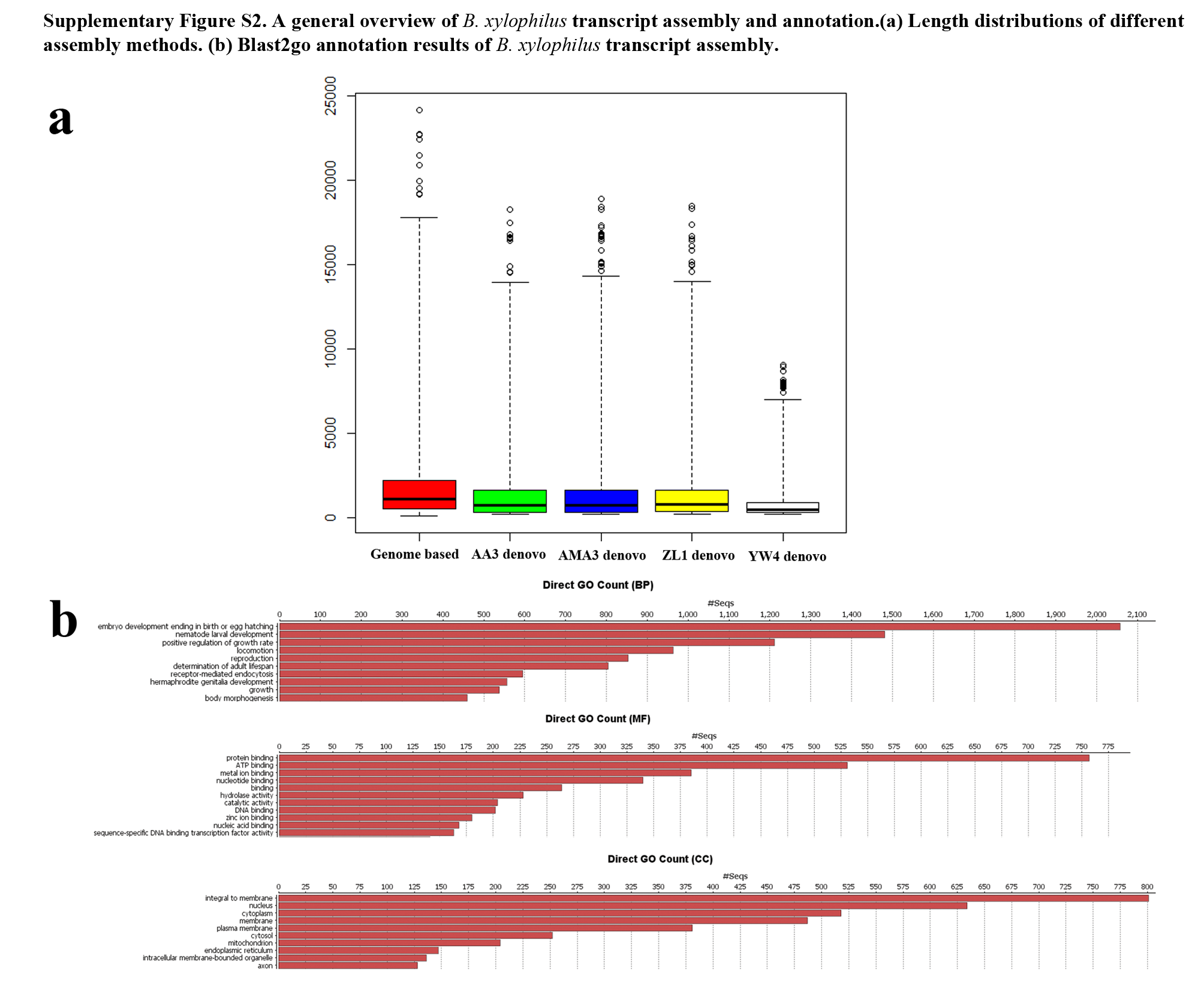

Supplement: S2 Fig — (a) Length distributions of different assembly methods. (b) Blast2go annotation results of B. xylophilus transcript assembly. (TIF) [file pone.0156040.s002.tif]

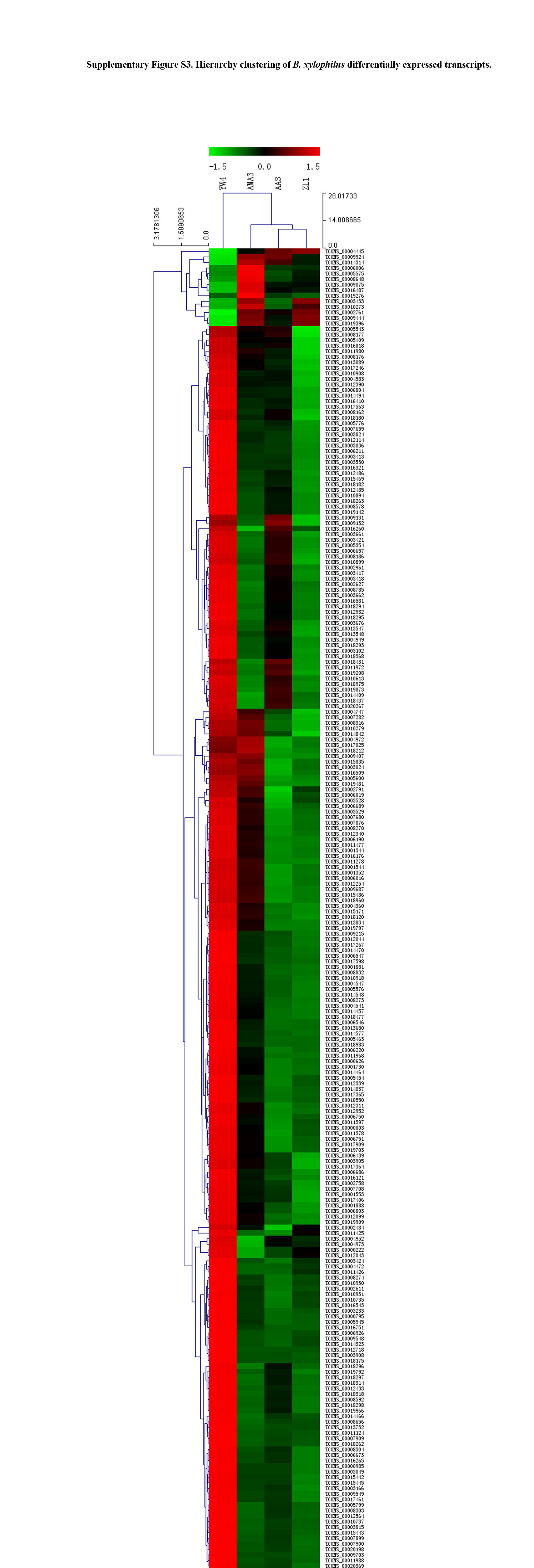

Supplement: S3 Fig — (TIF) [file pone.0156040.s003.tif]

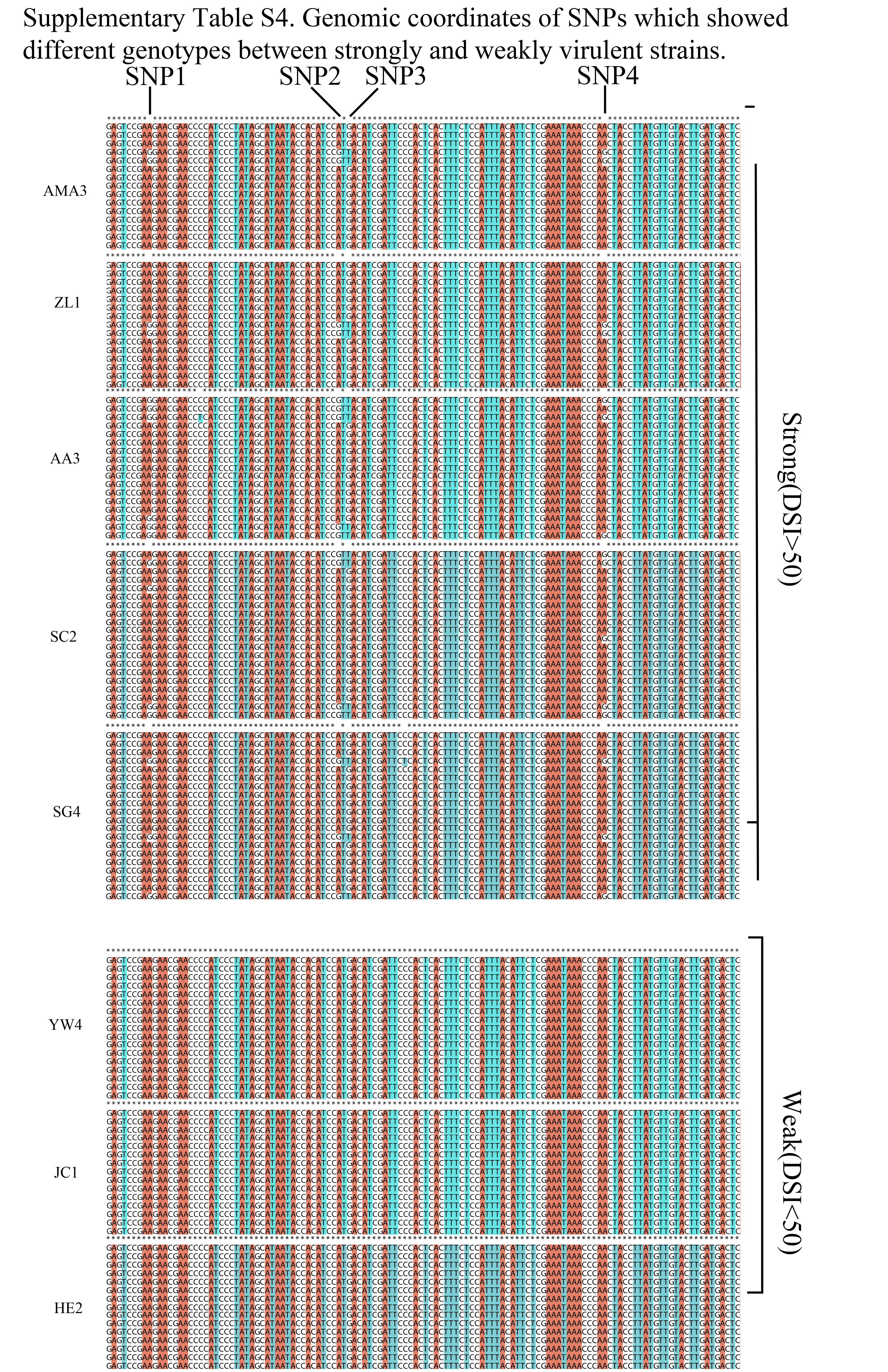

Supplement: S4 Fig — (TIF) [file pone.0156040.s004.tif]
