# Supplementary material for: Deciphering the Molecular Variations of Pine Wood Nematode Bursaphelenchus xylophilus with Different Virulence
Source: PLoS One. 2016 May 25;11(5):e0156040. doi: 10.1371/journal.pone.0156040 (PMC4880305; doi:10.1371/journal.pone.0156040)
Supplement: S2 Table — (PDF) [file pone.0156040.s006.pdf]

Supplementary Table S2 Detailed information of differentially expressed exons generated by DEXSeq.

DEXSeq differential exon usage test

Experimental design

sample	condition
control_YW4.count	control
treatment1_AA3.count	treatment
treatment2_AMA3.count	treatment
treatment3_ZL1.count	treatment

formulaDispersion = ~sample + exon + condition:exon

formula0 = ~sample + exon

formula1 = ~sample + exon + condition:exon

testForDEU result table

geneID	chr	start	end	total_exons	exon_changes
XLOC_000521 XLOC_000520 XLOC_000517 XLOC_000518	gi|351001753|emb|CADV01010257.1|	3433	16810	38	1
XLOC_000623	gi|351001765|emb|CADV01010245.1|	62156	64889	3	2
XLOC_000709 XLOC_000714	gi|351001785|emb|CADV01010225.1|	11357	18027	20	1
XLOC_001994	gi|351002454|emb|CADV01009556.1|	493	2762	5	1
XLOC_002687 XLOC_002692	gi|351002689|emb|CADV01009321.1|	1769	5549	10	6
XLOC_002961 XLOC_002967	gi|351002822|emb|CADV01009188.1|	9616	11917	6	1
XLOC_003208 XLOC_003204	gi|351002943|emb|CADV01009067.1|	15730	21492	14	6
XLOC_003401	gi|351003026|emb|CADV01008984.1|	26146	28035	7	1
XLOC_003991 XLOC_003990	gi|351003270|emb|CADV01008740.1|	7241	8219	7	1
XLOC_004276 XLOC_004280 XLOC_004281	gi|351003344|emb|CADV01008666.1|	2765	12517	33	1
XLOC_005135 XLOC_005139	gi|351003546|emb|CADV01008464.1|	3288	7913	17	1
XLOC_005292 XLOC_005286	gi|351003570|emb|CADV01008440.1|	4491	9608	16	6

XLOC_005377 XLOC_005372	gi|351003586|emb|CADV01008424.1|	2930	6996	7	1
XLOC_005426	gi|351003594|emb|CADV01008416.1|	372	11328	27	1
XLOC_006174 XLOC_006175 XLOC_006176	gi|351003867|emb|CADV01008143.1|	2556	5808	10	2
XLOC_006337 XLOC_006333	gi|351003935|emb|CADV01008075.1|	6166	13648	12	1
XLOC_007374 XLOC_007372	gi|351004472|emb|CADV01007538.1|	3210	6081	8	1
XLOC_007637	gi|351004647|emb|CADV01007363.1|	13150	13486	2	2
XLOC_008150	gi|351004855|emb|CADV01007155.1|	10368	19895	14	3
XLOC_008855 XLOC_008856	gi|351005050|emb|CADV01006960.1|	21122	27490	13	1
XLOC_009054 XLOC_009053	gi|351005162|emb|CADV01006848.1|	12512	15950	13	1
XLOC_009286 XLOC_009288	gi|351005313|emb|CADV01006697.1|	4384	10661	18	3
XLOC_009669 XLOC_009672	gi|351005524|emb|CADV01006486.1|	3569	16447	30	1
XLOC_009795 XLOC_009793	gi|351005553|emb|CADV01006457.1|	18511	26945	12	1
XLOC_011681	gi|351006228|emb|CADV01005782.1|	19917	23205	12	1
XLOC_011905	gi|351006313|emb|CADV01005697.1|	14693	15971	3	1
XLOC_011939	gi|351006324|emb|CADV01005686.1|	12192	19626	17	1
XLOC_012596 XLOC_012595	gi|351006577|emb|CADV01005433.1|	322	18836	22	2
XLOC_012669	gi|351006595|emb|CADV01005415.1|	71411	81930	22	2
XLOC_012887 XLOC_012890	gi|351006656|emb|CADV01005354.1|	27174	31363	7	1
XLOC_012927 XLOC_012915 XLOC_012914 XLOC_012928	gi|351006661|emb|CADV01005349.1|	77582	84005	29	1
XLOC_014450 XLOC_014448	gi|351007240|emb|CADV01004770.1|	11279	19727	20	5
XLOC_014530 XLOC_014523	gi|351007258|emb|CADV01004752.1|	13	12045	32	13
XLOC_014533 XLOC_014524	gi|351007258|emb|CADV01004752.1|	15234	25800	29	3
XLOC_014644 XLOC_014656	gi|351007275|emb|CADV01004735.1|	66124	72607	19	1
XLOC_015087 XLOC_015089 XLOC_015090	gi|351007416|emb|CADV01004594.1|	4408	8319	14	3

XLOC_015257 XLOC_015250	gi|351007479|emb|CADV01004531.1|	10166	12435	7	2
XLOC_015319 XLOC_015313	gi|351007503|emb|CADV01004507.1|	2975	8135	11	1
XLOC_015615 XLOC_015610	gi|351007549|emb|CADV01004461.1|	11650	15737	9	1
